# Associations between childhood and adulthood socioeconomic position and grip strength at age 46 years: findings from the 1970 British Cohort Study

**DOI:** 10.1186/s12889-022-13804-7

**Published:** 2022-07-27

**Authors:** Mohamed Yusuf, Gallin Montgomery, Mark Hamer, Jamie McPhee, Rachel Cooper

**Affiliations:** 1 Department of Sport and Exercise Sciences, Musculoskeletal Science and Sports Medicine Research Centre, Manchester Metropolitan University, 99 Oxford Road, Manchester, M1 7EL UK; 2 Manchester Metropolitan University Institute of Sport, Manchester, UK; 3 Institute of Sport, Exercise & Health, Division of Surgery & Interventional Science, University College London, 170 Tottenham Court Road, London, W1T 7HA UK; 4 AGE Research Group, Translational and Clinical Research Institute, Faculty of Medical Sciences, Newcastle University, Newcastle upon Tyne, UK; 5 NIHR Newcastle Biomedical Research Centre, Newcastle University and Newcastle Upon Tyne Hospitals NHS Foundation Trust, Newcastle upon Tyne, UK

**Keywords:** Grip strength, Muscle weakness, Socioeconomic position, Life course, Birth cohorts

## Abstract

**Background:**

Muscle weakness is a key criterion for important age-related conditions, including sarcopenia and frailty. Research suggests lower childhood socioeconomic position (SEP) may be associated with muscle weakness in later life but there is little evidence on associations in younger adults closer to peak muscle strength. We aimed to examine relationships between indicators of SEP in childhood and adulthood and grip strength at age 46y.

**Methods:**

We examined 7,617 participants from the 1970 British Cohort Study with grip strength measurements at 46y. We used sex-specific linear regression models to test associations between five different indicators of SEP in childhood and adulthood (paternal occupational class and parental education levels at age 5 and own occupational class and education level at age 46) and maximum grip strength. Models were adjusted for birth weight, BMI in childhood and adulthood, adult height, disability in childhood, leisure-time physical activity in childhood and adulthood, sedentary behaviour in childhood and adulthood, occupational activity and smoking at age 46.

**Results:**

Among women, lower SEP in childhood and adulthood was associated with weaker grip strength even after adjustments for covariates. For example, in fully-adjusted models, women whose mothers had no qualifications at age five had mean grip strength 0.99 kg (95% CI: -1.65, -0.33) lower than women whose mothers were educated to degree and higher. Among men, lower levels of father’s education and both adult SEP indicators were associated with stronger grip. The association between own occupational class and grip strength deviated from linearity; men in skilled-manual occupations (i.e. the middle occupational group) had stronger grip than men in the highest occupational group (Difference in means: 1.33 kg (0.60, 2.06)) whereas there was no difference in grip strength between the highest and lowest occupational groups. Adjustment for occupational activity largely attenuated these associations.

**Conclusion:**

Findings highlight the need to identify age and sex-specific interventions across life to tackle inequalities in important age-related conditions related to weakness.

**Supplementary Information:**

The online version contains supplementary material available at 10.1186/s12889-022-13804-7.

## Research summary

### What is already known on this subject?

• Muscle weakness (often indicated by low grip strength) is a key criterion for important age-related conditions including sarcopenia and frailty. It is highly prevalent in later life and can result from poor muscle development in earlier life and/or faster rates of age-related decline in strength from midlife.
• A growing body of evidence has shown that weak grip strength in later life may originate in early life and be influenced by factors including childhood and adulthood socioeconomic position (SEP).
• Most studies that have examined the association between early life SEP and grip strength have focused on older adults, and in the few studies that have examined younger adults findings are inconsistent.

### What this study adds?

• In a relatively large, nationally representative population of middle-aged adults in Great Britain we found sex differences in the associations between SEP and grip strength.
• In women, lower SEP in childhood and adulthood was consistently associated with weaker grip strength at age 46y.
• In men, there were no evidence of an association between two indicators of childhood SEP (father’s occupational class and mother’s education) and grip strength at age 46y. However, lower father’s educational attainment and lower adult SEP were associated with stronger grip, largely explained by higher levels of occupational activity in the skilled manual occupational group.

## Background

Muscle weakness, commonly indicated by low grip strength, is associated with mobility disability, loss of independence, premature mortality and many other adverse health outcomes [1–6]. It is also a key criterion for important age-related conditions including sarcopenia and frailty [7, 8]. These age-related conditions which are highly prevalent [9, 10] have profound implications for individuals, their families and society. In addition, estimates of the annual healthcare costs associated with muscle weakness and sarcopenia in a range of different countries around the world are substantial [11] and likely to increase with time as the global population ages. To address the public health challenge that muscle weakness represents we need to identify strategies that improve people’s chances of developing optimal strength in early life, maintaining strength through midlife and minimising decline in later life. This requires a better understanding of the risk factors across life that are associated with grip strength at different life stages.

Over the last two decades, a growing body of evidence has shown that differences in levels of grip strength in later life may originate in early life [12, 13]. This has resulted in investigations into the associations of various childhood factors with grip strength in adulthood, including indicators of socioeconomic position (SEP) [14, 15]. However, despite a systematic review published in 2011 that synthesised data from 12 studies on the association between childhood SEP and adult grip strength [14], and several subsequent investigations [15–23], evidence of an association between lower childhood SEP and weaker grip strength in adulthood remains equivocal. The authors of the systematic review reported considerable heterogeneity between studies [14]. This may be due to variations in the scale and direction of associations between childhood SEP and grip strength by age, sex, birth cohort, and/or place.

As most existing studies of childhood SEP and grip strength have focused on adults aged 60 and over [14, 16–19, 21–23], it is difficult to establish how associations vary across adulthood. In addition, even where existing studies have examined populations spanning a wide age range, including younger adults [18–22], interactions between age and SEP have rarely been formally tested [24]. Where associations have been observed between low childhood SEP and weak grip strength, it has not been possible to establish whether these are explained by the influences of SEP in early life on the attainment of peak grip strength or its subsequent decline. More studies of younger adults closer to peak grip strength are required to establish this. This is especially as the only study on younger adults, included in the systematic review [14], found lower childhood SEP was associated with stronger grip in Swedish males at age 18. This is in the opposite direction to the association reported in some studies of older adults highlighting that childhood SEP may have different patterns of association with grip strength at different life stages.

Also limiting our understanding of childhood SEP and grip strength associations is the fact that most studies only include adults born before 1950 [15–23]. Whether similar associations are also found in more recently born generations exposed to different social, political, economic and work environments across life also remains to be established.

To address the need for studies of the association between SEP and grip strength in younger adults from more recently born cohorts, we aimed to explore the relationships between indicators of SEP in childhood and adulthood with grip strength at age 46y in the 1970 British Cohort Study. We examined: (a) whether indicators of SEP prospectively ascertained in childhood and adulthood were associated with grip strength; (b) whether these associations varied by sex and were explained by several important covariates.

## Methods

### Study design and population

**Fig. 1:**
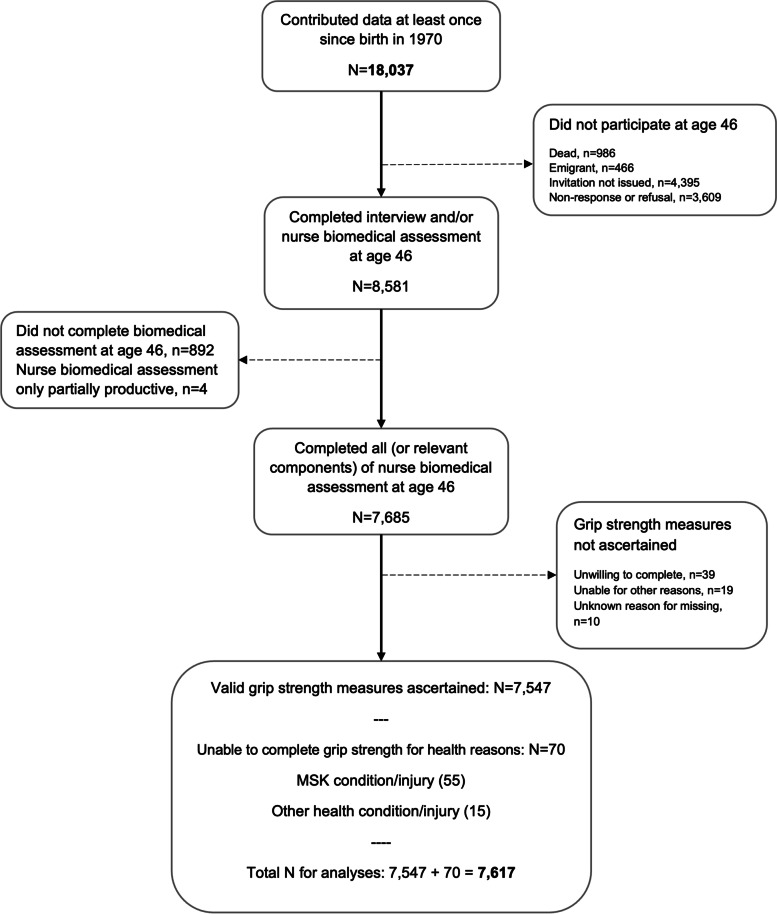
Flow diagram of participation in the BCS70

We conducted secondary analysis using data from the 1970 British Cohort Study (BCS70), an ongoing prospective study of males and females born in England, Scotland and Wales within a single week in March 1970, with immigrants added into the sample during the first three waves [25]. A total of 18,037 males and females were recruited and assessed on at least one occasion (at birth, and ages 5, 10, 16, 26, 30, 34, 38, 42 and 46) [26]. At age 46, a home visit was conducted, during which a 50-min interview and a nurse-led biomedical assessment, including grip strength measurement, was undertaken. A total of 8,581 participants completed at least one component of the data collection at age 46 (Fig. 1). Of these, 7,685 completed a nurse biomedical assessment, and 7,547 had valid grip strength measures. Participants provided informed consent and the assessment at age 46y received full ethical approval from NRES Committee South East Coast—Brighton & Sussex (Ref 15/LO/1446).

### Assessment of grip strength

During the biomedical assessment at age 46y, grip strength was measured in kilograms using a Smedley hand-held dynamometer by trained nurses following standardised protocols. The maximum measurement of six attempts (three in each hand) was used in analyses. Participants were excluded from the grip strength assessment if they had had hand surgery in the past six months or had swelling, inflammation, severe pain, or a recent injury to their hands. If participants were unable or unwilling to complete the grip strength tests, the reason for this was recorded. Participants unable to complete the grip strength assessment for health reasons (*n* = 70) were allocated a value of grip strength equivalent to the mean of the bottom sex-specific fifth of the grip strength distribution [17] on the assumption that these participants were likely to have had low grip strength whereby their exclusion may bias results [27].

### Socioeconomic position

We chose a priori to use indicators of SEP ascertained at ages five and 46y. At age five, we used father’s occupational class (or at birth if missing (*n* = 1,176)) and mother’s and father’s educational levels. Using the Registrar General’s Social Classification (RGSC), occupational class was categorised into four groups: I professional/II intermediate, III skilled non-manual, III skilled manual and IV partly skilled/V unskilled. Both mother’s and father’s educational levels were based on the highest qualification achieved categorised into four groups: Higher vocational/degree and higher, A-level/equivalent (advanced secondary education), Vocational/O-level/equivalent (ordinary secondary education) and No qualification. At age 46, we selected to use own occupational class, back-coded from National Statistics Socio-Economic Classification of occupations to RGSC, and then similarly categorised as father’s occupational class. Own highest qualification at age 46 was also used, categorised into four groups: Degree and higher, A-level and vocational qualification (advanced secondary education), GCSEs (ordinary secondary education) and no qualifications.

### Covariates

Covariates were selected a priori based on previous literature [28, 29] and considered within the framework outlined in supplementary Figure S1. As height is strongly associated with grip strength [28], and in many cases, relative grip strength (i.e., grip strength adjusted for height) is presented as a primary outcome measure [30], analyses were initially adjusted for adult *height* (nurse-measured at age 46). Childhood factors included: birth weight (kg) (ascertained from birth records) and the following variables assessed at age ten: body mass index (BMI) (calculated as kg/m^2^ from nurse-measured height and weight); leisure-time physical activity (maternal report of how often the participant played sports in their spare time); sedentary behaviour (maternal report of how often the participant watched television in their spare time); disability (parental report of whether they considered the participant to have a physical or mental disability or handicap, or any other disabling condition which interfered with everyday life, or which might be a problem at school).

Adulthood covariates were BMI at age 46 (derived from nurse-measured height and weight); self-reported smoking status at age 42; sedentary behaviour at age 42 (self-reported length of time spent watching television on a typical weekday); leisure-time physical activity at age 42 (self-reported number of days spent doing 30 min or more of exercise in a typical week); occupational activity at age 46 (self-report of the types of physical activity involved in the participant’s work). The categorisation of all covariates are presented in Table 1.

**Table 1:** Characteristics of the BCS70 participants included in analyses (maximum *N* = 7,617*)

		**Mean (SD) or N (%) **^**a**^		
**Characteristics**	**Overall N **^**a**^	**Female** (*N*** = **3,922)	**Male** (*N* = 3,695)	***p*-value **^**b**^
**Outcome**				
Max grip strength (kg) at age 46y ^c^	7,617	29.51 (5.83)	48.03 (9.01)	< 0.001
**Socioeconomic indicators**				
Father's occupational class at age 5y	7,198			0.07
* I Professional/II Intermediate*		1,059 (28.4)	1,039 (29.9)	
* III Skilled non-manual*		399 (10.7)	390 (11.3)	
* III Skilled manual*		1,584 (42.5)	1,479 (42.6)	
* IV Partly skilled/V Unskilled*		686 (18.4)	562 (16.2)	
Mother's highest qualification at age 5y	5,931			0.96
* Higher vocational/degree and higher*		289 (9.4)	276 (9.7)	
* A-level/equivalent*		138 (4.5)	134 (4.7)	
* Vocational/O-level/equivalent*		1,095 (35.5)	1,010 (35.4)	
* No qualification*		1,557 (50.6)	1,432 (50.2)	
Father's highest qualification at age 5y	5,565			0.74
* Higher vocational/degree and higher*		520 (18.1)	512 (19.0)	
* A-level/equivalent*		253 (8.8)	221 (8.2)	
* Vocational/O-level/equivalent*		845 (29.5)	789 (29.2)	
* No qualification*		1,252 (43.6)	1,173 (43.5)	
Own occupational class at age 46y	6,404			< 0.001
* I Professional/II Intermediate*		1,410 (45.4)	1,614 (48.9)	
* III Skilled non-manual*		997 (32.1)	469 (14.2)	
* III Skilled manual*		326 (10.5)	959 (29.1)	
* IV Partly skilled/V Unskilled*		372 (12.0)	257 (7.8)	
Own highest qualification at age 46y	7,512			< 0.001
* Degree and higher*		1,055 (27.2)	923 (25.4)	
* A-level and vocational qualification*		608 (15.7)	467 (12.9)	
* GCSEs*		1,243 (32.1)	1,119 (30.8)	
* No qualification*		973 (125.)	1,124 (31.0)	
**Covariates**				
Birth weight (kg) (Mean (SD))	7,046	3.26 (0.50)	3.37 (0.53)	< 0.001
BMI (kg/m^2^) at age 10y (Mean (SD))	6,016	16.93 (2.20)	16.73 (1.90)	0.025
Leisure-time physical activity at age 10y	6,607			< 0.001
* Never or hardly ever*		365 (10.7)	158 (4.9)	
* Sometimes*		1,643 (48.3)	920 (28.7)	
* Often*		1,392 (40.9)	2,129 (66.4)	
Sedentary behaviour (TV watching) at age 10y	6,626			< 0.001
* Never or hardly ever*		49 (1.4)	26 (0.8)	
* Sometimes*		810 (23.7)	512 (15.9)	
* Often*		2,559 (74.8)	2,670 (83.3)	
Disability at age 10y	6,606			0.057
* No*		3,203 (93.7)	2,943 (92.3)	
* Yes, slight*		195 (5.7)	228 (7.1)	
* Yes, severe*		19 (0.6)	18 (0.6)	
Height (m) at age 46y (Mean (SD))	7,553	1.64 (0.06)	1.77 (0.07)	< 0.001
BMI (kg/m^2^) at age 46y (Mean (SD))	7,387	28.22 (6.17)	28.64 (4.63)	< 0.001
Smoking status at age 42y	7,111			0.003
* Never smoker*		1,776 (48.0)	1,590 (46.6)	
* Ex-smoker*		1,111 (30.0)	966 (28.3)	
* Current smoker (less than daily)*		210 (5.7)	183 (5.4)	
* Current smoker (daily)*		604 (16.3)	671 (19.7)	
Sedentary behaviour (TV watching) at age 42y	6,368			< 0.001
* 0 to* < *1 h*		624 (18.4)	447 (15.0)	
* 1 to* < *3 h*		1,976 (58.4)	1,790 (60.0)	
* 3 to* < *5 h*		621 (18.4)	555 (18.6)	
* 5* + *hours*		162 (4.8)	193 (6.5)	
Leisure-time physical activity (days/week) at age 42y	7,008			< 0.001
* 0 days*		1,135 (31.1)	809 (24.0)	
* 1 day per a week*		438 (12.0)	390 (11.6)	
* 2 days per a week*		560 (15.4)	482 (14.3)	
* 3 days per a week*		534 (14.6)	506 (15.1)	
* 4/5 days per a week*		549 (15.1)	661 (19.7)	
* 6/7 days per a week*		430 (11.8)	514 (15.3)	
Occupational activity at age 46y	6,291			< 0.001
* Sitting occupation*		1,756 (55.7)	1,682 (53.6)	
* Standing occupation*		635 (20.1)	337 (10.7)	
* Physical work*		733 (23.3)	850 (27.1)	
* Heavy manual work*		28 (0.9)	270 (8.6)	

^*^ Sample restricted to those with valid measures of grip strength at age 46y. Including those who were unable to complete the grip strength for health reasons whose values have been imputed a value of grip strength equivalent to the mean of the bottom sex-specific fifth of the grip strength distribution (*N* = 70)

*N* = Total number

^a^ Ns presented in table vary due to missing data

^b^ Statistical tests of sex difference: chi-square of independence; t-test

^c^ Including those 70 people with imputed grip strength values. Observed mean max grip strength (*N* = 7,547): Females 29.60 kg (SD 5.81 kg); Males 48.09 kg (SD 8.99 kg)

### Statistical analyses

T-tests and chi-squared tests were used to examine sex differences in continuous and categorical variables, respectively. We tested the associations between each SEP indicator and maximum grip strength at 46y using linear regression models. We first ran formal tests of interaction between sex and each SEP indicator and where there was evidence of sex interaction (based on *p* < 0.1) subsequent models were stratified by sex. Linear trends were assessed using likelihood ratios tests. Covariates were added to the models sequentially. Initially, adult height was included, then childhood factors, and then adulthood factors. As well as running models with factors grouped (as presented) we also examined associations with adjustment for each adulthood covariate added in turn. To take account of the continuity of SEP from childhood to adulthood, associations between childhood SEP indicators and grip strength were also adjusted for adulthood SEP.

To reduce selection bias, we used sex-stratified multiple imputation, which assumes that the data were missing at random, with chained equations to impute missing values in the explanatory factors and covariates (missing data ranged from 0.8% (height at 46y) to 26.9% (father’s education) – see Table S1 and S2 in Additional file 1) in the sample with valid data on grip strength (including those 70 individuals unable for health reasons with imputed values) (*N* = 7,617) (Fig. 1) [31]. As a larger number of imputations have been suggested in settings where high statistical power is needed, we utilised 50 imputations [32]. Analyses were run on the 50 imputed data sets created, and estimates were combined using Rubin’s rules [33]. All analyses were conducted using R (R Foundation for Statistical Computing, v4.0.3, Vienna, Austria).

### Sensitivity analyses

Sensitivity analyses were conducted to check that the results were not influenced by: 1) inclusion of participants who completed the grip strength assessment sat down or with their arm supported (*N* = 727); 2) inclusion of participants who were unable to complete their grip strength assessment due to health reasons (*N* = 70); 3) inclusion of participants who reported disability at age 46 according to the European Statistics of Income and Living Conditions classification (severely hampered (*n* = 452) or missing information disability (*N* = 3)).

## Results

Table 1 shows the distributions of childhood and adulthood characteristics by sex in the analytic sample. Men had higher mean grip strength at age 46 than women (48.0 kg vs 29.5 kg; *p* < 0.001). Participants were most often born to fathers with occupational class III manual and parents with no formal qualifications.

**Fig. 2:**
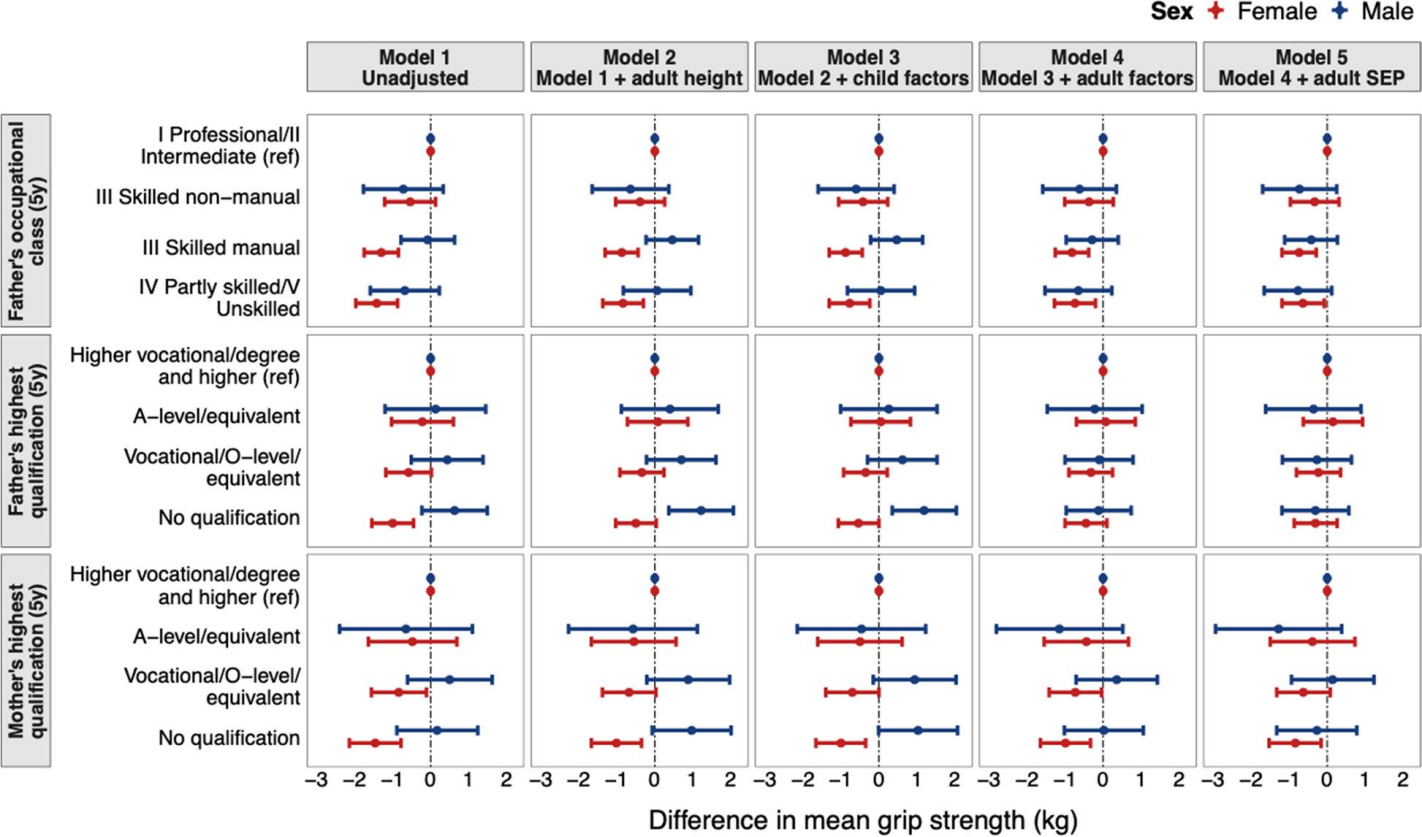
Associations between indicators of childhood socioeconomic position and grip strength at age 46 years in the BCS70 (linear regression models stratified by sex with sample restricted to those with valid measures of grip strength at age 46 years (maximum *N* = 7,617* (3,922 females and 3,695 males))) results are combined from analyses run across 50 imputed datasets. *70 participants unable to complete the grip strength tests for health reasons were included by allocating them grip strength values equivalent to the mean of the bottom sex-specific fifth. Model 1: unadjusted (p-values from formal tests of sex interaction, *p* = 0.015 for Father’s occupation at age 5y, *p* = 0.025 for Mother’s highest qualification at age 5y and *p* = 0.016 for Father’s highest qualification at age 5y); Model 2: adjusted for height at age 46y; Model 3: adjusted for Model 2 + birth weight (kg), BMI at age 10y (kg/m^2^), leisure-time physical activity at age 10y, sedentary behaviour (TV watching) at age 10y and disability at age 10y; Model 4: adjusted for Model 3 + BMI at age 46y (kg/m^2^) + smoking status at age 42y, sedentary behaviour (TV watching) at age 42y, leisure-time physical activity (days/week) at age 42y and occupational activity at age 46y

**Fig. 3:**
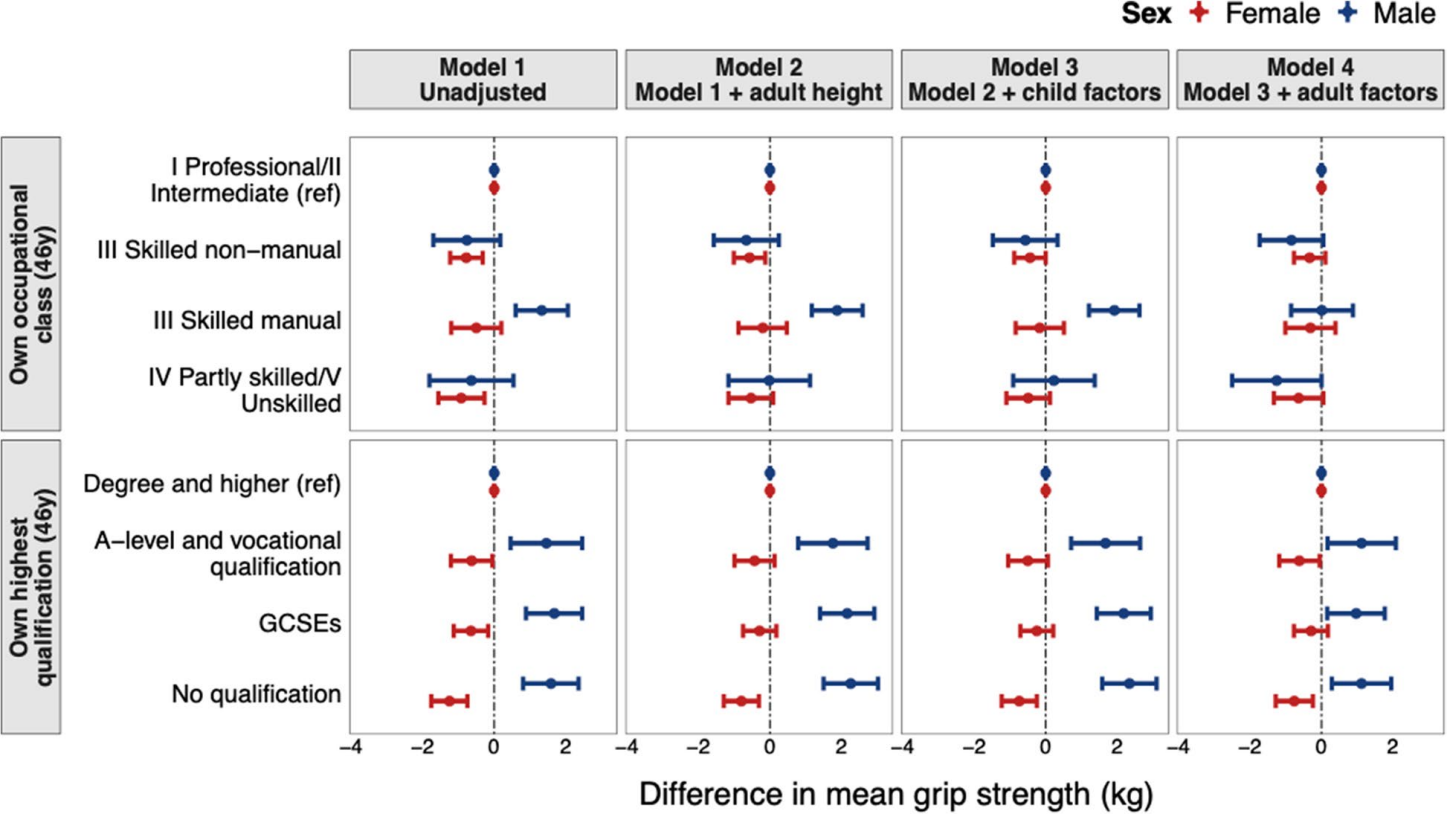
Associations between indicators of adulthood socioeconomic position and grip strength at age 46 years in the BCS70 (linear regression models stratified by sex with sample restricted to those with valid measures of grip strength at age 46 years (maximum *N* = 7,617* (3,922 females and 3,695 males))) results are combined from analyses run across 50 imputed datasets. *70 participants unable to complete the grip strength tests for health reasons were included by allocating them grip strength values equivalent to the mean of the bottom sex-specific fifth. Model 1: unadjusted (p-values from formal tests of sex interaction, *p* = 0.015 for Fathers occupation at age 5y, *p* = 0.025 for Mother’s highest qualification at age 5y and *p* = 0.016 for Father’s highest qualification at age 5y); Model 2: adjusted for height at age 46y; Model 3: adjusted for Model 2 + birth weight (kg), BMI at age 10y (kg/m^2^), leisure-time physical activity at age 10y, sedentary behaviour (TV watching) at age 10y and disability at age 10y; Model 4: adjusted for Model 3 + BMI at age 46y (kg/m^2^) + smoking status at age 42y, sedentary behaviour (TV watching) at age 42y, leisure-time physical activity (days/week) at age 42y and occupational activity at age 46y

Figures 2 and 3, and Tables S3 and S4 in Additional file 1, show the sex-stratified associations between childhood and adulthood SEP indicators and grip strength. All associations between childhood and adulthood SEP indicators and grip strength varied by sex (*p*_interaction_ < 0.05) (Tables S3 and S4 in Additional file 1**)**. Among women, in unadjusted analyses, lower SEP was linearly associated with weaker grip strength (*p* < 0.001) (Figs. 2 and 3, Tables S3 and S4); this was observed for all five SEP indicators. When models of the associations between the three indicators of childhood SEP and grip strength were adjusted for covariates, associations of lower father’s occupational class and lower mother’s education with weaker grip strength were partly attenuated but even after adjustment for adult SEP, modest associations remained (Fig. 2). For example, in the unadjusted model, women whose mothers had no qualifications had mean grip strength 1.46 kg (95% CI: -2.14, -0.78) lower than women whose mothers were educated to vocational/degree or higher, and in the fully adjusted model, this difference was 0.99 kg (-1.65, -0.33). However, associations between lower father’s education and weaker grip strength were fully attenuated after adjustment for adult height. In adulthood, the association of lower educational attainment and weaker grip strength was partially attenuated after adjustments, but an association remained in the final model (Fig. 3, Table S4). In contrast, the association between lower own occupational class and weaker grip strength was fully attenuated after adjustment for adult height (Fig. 3).

In men, there was no evidence of association between father’s occupational class or mother’s education and grip strength (Fig. 2, Table S3). However, in height-adjusted models, there was evidence that lower father’s education was associated with stronger grip. This association was maintained after adjustment for childhood factors but fully attenuated after adjustment for adulthood factors (Fig. 2). Lower SEP in adulthood was also associated with stronger grip at age 46 (Fig. 3, Table S4). The association between lower own education and stronger grip was strengthened after adjustment for height and childhood covariates and only partially attenuated after adjustment for adulthood covariates.

The association between own occupational class and grip strength in men deviated from linearity (Fig. 3, Table S4); men in skilled manual occupations had a stronger grip than men in professional/intermediate occupations (unadjusted regression coefficient: 1.33 kg (0.60, 2.06)) whereas there was no difference in grip strength between the highest and lowest occupational groups. This association strengthened after adjustment for height and childhood factors, but inclusion of adulthood factors fully attenuated the association (0.01 kg (-0.85, 0.88)). The attenuation of associations in men after adjustment for adult factors was found to be largely driven by occupational activity (Table S5).

Results from models run on complete cases were similar to those run across imputed datasets (Table S6). Results from sensitivity analyses were similar to those described above, suggesting that our analyses are unlikely to be impacted by the: 1) inclusion of participants who completed grip strength assessment sat down or with the arm supported (Table S7); 2) inclusion of participants who were unable to complete their grip strength assessment due to health reasons (Table S8); 3) the inclusion of participants who were severely hampered or had missing information on disability (Table S9).

## Discussion

### Main findings

In a cohort of men and women followed from birth in 1970 until midlife, we found sex-specific associations between SEP in childhood and adulthood and grip strength at age 46y. In women, there was evidence of associations between all five indicators of SEP and weaker grip strength, and many of these, including associations of father’s occupational class and mother’s education with grip strength, were robust to adjustments for body size and childhood and adulthood factors. Notably, associations between childhood SEP and grip strength were not fully explained by the continuity of SEP between childhood and adulthood. In men, there was no evidence of an association between two of the three childhood indicators of SEP and grip strength. However, lower father’s education (once height-adjusted) and lower adulthood SEP were associated with stronger grip. The non-linear association for own occupational class was explained by higher levels of occupational activity among men in skilled manual occupations.

### Comparison with previous studies

Our work adds to existing literature on associations between childhood and adulthood SEP and grip strength. We found evidence of associations between childhood and adulthood SEP and weaker grip strength in women. In previous studies, where associations have been observed, lower SEP was typically associated with weaker grip strength and so our findings in women are consistent [16, 18–22]. However, our study is the first to show robust associations between prospectively ascertained childhood and adulthood SEP and grip strength in middle-aged women. Our findings of associations between lower adulthood SEP and stronger grip in men contrast with what has been reported in many previous studies in older adults [14, 16–23]. It is also not fully consistent with analyses of the UK Household Longitudinal Study (UKHLS) which reported that in men there was no overall association between educational attainment and grip strength and associations between lower maternal education, lower income and weaker grip [24]. However, in UKHLS, participants were aged 16 to 99y, and so the authors were able to test variation by age. When they did this there was some evidence of an association between lower SEP and stronger grip in earlier adulthood. This is consistent with our findings and another study of younger men—an association between lower SEP and stronger grip was reported in a study of Swedish male military personnel aged 18y [34]. Our findings thus add further weight to the suggestion that in men, associations between SEP and grip strength may change direction with age.

### Explanation of findings

In considering potential explanations of the consistent associations between lower childhood and adulthood SEP and weaker grip in women, it is necessary to consider the different factors that could be acting on pathways between SEP across life and grip strength in midlife. This is because SEP indicators are distal factors, and therefore associations would be expected to be mediated by more proximal factors that are socioeconomically patterned and relate to subsequent grip strength. In identifying these factors, it is important to consider the complex biological and social pathways that have been proposed to explain socioeconomic differences in health outcomes [35]. In the case of grip strength, there are likely to be a range of factors and pathways implicated including those related to growth and development (both in utero and across childhood and adolescence), and the factors that drive this (i.e. nutrition and exposure to hormones), attainment of adult body size and composition, health behaviours (most importantly physical activity) and health status [29]. Although we were able to adjust for some of these factors in our analyses, there are others that we were not able to, such as differences in sex hormones and diet across life.

While similar pathways are likely to operate in men, our findings suggest that occupational activity (specifically physical and heavy manual work) is countering some of the potentially adverse effects of low SEP on grip strength in midlife. As occupational activity was the factor in adulthood that caused the greatest attenuation in the scale of the associations between father’s education and both indicators of adult SEP and grip strength, this would suggest that occupational activity may be responsible for the associations we observe in men. This is consistent with findings from a study of Danish men with a mean age of 59y which reported an association between higher levels of specific types of occupational activity and stronger grip [36]. Occupational activity has historically been linked with premature mortality [37]; however, a recent nationwide prospective cohort study in Norway found a positive dose–response relationship between occupational activity and longevity in men that was explained by a range of covariates including body mass index, lifestyle factors, cardiovascular diseases, and childhood SEP [38], and an umbrella review prepared for the *2020 WHO Physical Activity Guideline Development Group* found occupational activity to protect against most health-related outcomes, including cancers, heart disease, and type 2 diabetes [39]. Our results are consistent with these studies suggesting potentially protective effects of occupational activity in middle-aged men.

### Methodological considerations

Our study addresses an important research gap by examining the associations between childhood and adulthood SEP and grip strength in younger adults from a more recently born cohort than previous studies. Another benefit of studying a younger cohort is that they are still relatively healthy and so the potential confounding effects of age-related health conditions which may explain associations in older populations are minimised. We formally tested sex differences in our associations which is important as it has previously been suggested that sex differences might be a potential source of variation between studies of the association between childhood SEP and grip strength [14].

Another key strength of our study is that we used a large, population-based sample that was nationally representative at birth with prospectively ascertained SEP indicators and several potentially important covariates from multiple time points. Nonetheless, as in all longitudinal studies, the BCS70 has experienced attrition, which may have introduced bias. BCS70 participants who contributed data at age 46y were more likely to be women, be taller, less likely to be current smokers and have a higher childhood and adulthood SEP than those lost to follow-up [26]. However, we maximised our analytic sample and minimised potential bias due to missing data by using multiple imputation. Here, we used sex-stratified multiple imputation to account for the sex-interactions in our associations [31]. By using multiple imputation, we were making the assumption that data were missing at random but, we have to acknowledge that this may not have been the case. However, we did use a range of auxiliary variables that are predictive of missingness within the BCS70 in our multiple imputation [40, 41]. Other limitations include the inability to examine associations between SEP and grip strength by ethnicity, as much of the BCS70 is white-British. This makes it difficult to fully generalise our findings to today’s population in Great Britain, although the BCS70 cohort does provide important new insights on the associations between childhood and adulthood SEP and grip strength that complement findings from older cohorts because of their exposure to more contemporaneous social and political factors.

Another potential limitation is that our study utilised a single question to measure occupational activity; future research would benefit from more detailed measurements of occupational activity including data on intensity, duration, and frequency of activity. We also acknowledge that there may be residual confounding. However, in our analyses we adjusted for a wide range of covariates.

### Policy implications

As low grip strength is associated with higher subsequent risk of disability [2], and reduces one’s chances of living a healthy, independent life, the findings reported in this study are meaningful in the context of the UK government’s ambitious goal of *‘ensuring that people can enjoy at least five extra healthy, independent years of life by 2035, while narrowing the gap between the experience of the richest and poorest’* [42]. They highlight the complexity of the associations between SEP and grip strength and the need to identify age and sex-specific interventions to tackle the stark health inequalities in important age-related conditions related to muscle weakness.

In women, as lower SEP in childhood and adulthood was associated with weaker grip strength, strategies to reduce their exposure to socioeconomic adversity across life are likely to benefit their grip strength at midlife. For men, lower SEP in adulthood was associated with stronger grip at age 46y, which was mostly attenuated by higher levels of occupational activity. Evidence from the oldest British birth cohort, born in 1946, suggests that an association between lower lifetime SEP and weaker grip emerges as the cohort age (there is limited evidence of association at age 53 but an association is seen at 60–64 and 69) [15, 17, 23]. This suggests that the association in BCS70 may change with age, especially as there is evidence that the protective effects of occupational activity may recede by the time of retirement [43, 44]. As this could relate to either reductions in levels of beneficial activity and/or the accumulation of wear and tear related to heavy manual work, further research is needed to identify the types of interventions that may be most effective in ensuring that men of lower SEP maintain any midlife strength advantage into later life.

## Conclusions

We have identified sex differences in the associations between childhood and adulthood SEP and grip strength in middle-aged British adults. Our findings highlight the need to identify age and sex-specific interventions to tackle inequalities across life in important age-related conditions related to weakness.

## Supplementary Information

**Additional file 1:** **Figure S1.** Pathway diagram detailing the illustrates the proposed pathways of association. **Table S1.** Variables used in multiple imputation**Table S2.** A comparison of the distributions of variables included in the multiple imputation models by completeness of data. **Table S3.** Associations between indicators of childhood socioeconomic position and grip strength at age 46 years in the BCS70 (linear regression models in A) Females (*n*=3,922) and B) Males (*n*=3,695)). **Table S4.** Associations between indicators of adulthood socioeconomic position and grip strength at age 46 years in the BCS70 (linear regression models in A) Females (*n*=3,922) and B) Males (*n*=3,695)). **Table S5.** Associations between adulthood socioeconomic position and grip strength at age 46 years in the BCS70 with individual adjustments for each adult covariate (linear regression models in Males (*n*=3,695)). **Table S6.** Unadjusted associations between indicators of childhood and adulthood socioeconomic position and grip strength at age 46 years in the BCS70 on observed data (linear regression models in A) Females (*n*=3,922) and B) Males (*n*=3,695)). **Table S7.** Associations between indicators of childhood and adulthood socioeconomic position and grip strength at age 46 years in the BCS70 on the sample who completed the grip strength assessment standing unsupported (linear regression models in A) Females (*n*=3,498) and B) Males (*n*=3,392))[Sensitivity analysis]. **Table S8.** Associations between indicators of childhood and adulthood socioeconomic position and grip strength at age 46 in the BCS70 excluding those participants unable to complete the grip strength assessments for health reasons(linear regression models in A) Females (*n*=3,872) and B) Males (*n*=3,675)) [Sensitivity analysis]. **Table S9.** Associations between indicators childhood and adulthood socioeconomic position and grip strength at age 46 in the BCS70 excluding those participants classified as severely hampered according to the European Statistics of Income and Living Conditions (EU-SILC) classification disability definition or with missing disability data (linear regression models in A) Females (*n*=3,638) and B) Males (*n*=3,394)) [Sensitivity analysis].

**Additional file 2:** 

## Data Availability

Data used in this paper are available from the UK Data Service and were accessed via a standard data application (project ID 186390). The datasets used were: Chamberlain, G., University of London, Institute of Education, Centre for Longitudinal Studies, Chamberlain, R. (2013). *1970 British Cohort Study: Birth and 22-Month Subsample, 1970–1972*.[data collection]. 3rd Edition. UK Data Service. SN: 2666, http://doi.org/10.5255/UKDA-SN-2666-2 University of London, Institute of Education, Centre for Longitudinal Studies, Osborn, A., Dowling, S., Butler, N. (2021). *1970 British Cohort Study: Age 5, Sweep 2 1975*.[data collection]. *5th Edition*. UK Data Service. SN: 2699, http://doi.org/10.5255/UKDA-SN-2699-4 University of London, Institute of Education, Centre for Longitudinal Studies, Butler, N., Bynner, J. (2021). *1970 British Cohort Study: Age 10, Sweep 3, 1980*.[data collection]. *6th Edition*. UK Data Service. SN: 3723, http://doi.org/10.5255/UKDA-SN-3723-7 University of London, Institute of Education, Centre for Longitudinal Studies. (2021). *1970 British Cohort Study: Age 46, Sweep 10, 2016–2018*.[data collection]. UK Data Service. SN: 8547, http://doi.org/10.5255/UKDA-SN-8547-1 University College London, UCL Institute of Education, Centre for Longitudinal Studies. (2021). *1970 British Cohort Study Response Dataset, 1970–2016*.[data collection]. *4th Edition*. UK Data Service. SN: 5641, http://doi.org/10.5255/UKDA-SN-5641-3 We are grateful to the Centre for Longitudinal Studies (CLS), UCL Social Research Institute, for the use of these data and to the UK Data Service for making them available.

## Abbreviations

BCS70: 1970 British Cohort Study
BMI: Body Mass Index
RGSC: Registrar General’s Social Classification
SEP: Socioeconomic position
UKHLS: UK Household Longitudinal Study
