# Supplementary material for: Associations between childhood and adulthood socioeconomic position and grip strength at age 46 years: findings from the 1970 British Cohort Study
Source: BMC Public Health. 2022 Jul 27;22:1427. doi: 10.1186/s12889-022-13804-7 (PMC9327373; doi:10.1186/s12889-022-13804-7)
Supplement: Supplementary file 1 — Additional file 1: Figure S1. Pathway diagram detailing the illustrates the proposed pathways of association. Table S1. Variables used in multiple imputationTable S2. A comparison of the distributions of variables included in the multiple imputation models by completeness of data. Table S3. Associations between indicators of childhood socioeconomic position and grip strength at age 46 years in the BCS70 (linear regression models in A) Females (n=3,922) and B) Males (n=3,695)). Table S4. Associations between indicators of adulthood socioeconomic position and grip strength at age 46 years in the BCS70 (linear regression models in A) Females (n=3,922) and B) Males (n=3,695)). Table S5. Associations between adulthood socioeconomic position and grip strength at age 46 years in the BCS70 with individual adjustments for each adult covariate (linear regression models in Males (n=3,695)). Table S6. Unadjusted associations between indicators of childhood and adulthood socioeconomic position and grip strength at age 46 years in the BCS70 on observed data (linear regression models in A) Females (n=3,922) and B) Males (n=3,695)). Table S7. Associations between indicators of childhood and adulthood socioeconomic position and grip strength at age 46 years in the BCS70 on the sample who completed the grip strength assessment standing unsupported (linear regression models in A) Females (n=3,498) and B) Males (n=3,392))[Sensitivity analysis]. Table S8. Associations between indicators of childhood and adulthood socioeconomic position and grip strength at age 46 in the BCS70 excluding those participants unable to complete the grip strength assessments for health reasons(linear regression models in A) Females (n=3,872) and B) Males (n=3,675)) [Sensitivity analysis]. Table S9. Associations between indicators childhood and adulthood socioeconomic position and grip strength at age 46 in the BCS70 excluding those participants classified as severely hampered according to the European Statist [file 12889_2022_13804_MOESM1_ESM.pdf]

Appendix Material for Online Publication: Associations between socioeconomic position across life and grip strength at age 46 years: findings from the 1970 British Cohort Study

Mohamed Yusuf, Gallin Montgomery, Mark Hamer, Jamie McPhee, Rachel Cooper

Figure S1: Pathway diagram detailing the illustrates the proposed pathways of association

Table S1: Variables used in multiple imputation

Table S2: A comparison of the distributions of variables included in the multiple imputation models by completeness of data

Table S3: Associations between indicators of childhood socioeconomic position and grip strength at age 46 years in the BCS70 (linear regression models in A) Females (n=3,922) and B) Males (n=3,695))

Table S4: Associations between indicators of adulthood socioeconomic position and grip strength at age 46 years in the BCS70 (linear regression models in A) Females (n=3,922) and B) Males (n=3,695))

Table S5: Associations between adulthood socioeconomic position and grip strength at age 46 years in the BCS70 with individual adjustments for each adult covariate (linear regression models in Males (n=3,695))

Table S6: Unadjusted associations between indicators of childhood and adulthood socioeconomic position and grip strength at age 46 years in the BCS70 on observed data (linear regression models in A) Females (n=3,922) and B) Males (n=3,695))

Table S7: Associations between indicators of childhood and adulthood socioeconomic position and grip strength at age 46 years in the BCS70 on the sample who completed the grip strength assessment standing unsupported (linear regression models in A) Females (n=3,498) and B) Males (n=3,392)) *[Sensitivity analysis]*

Table S8: Associations between indicators of childhood and adulthood socioeconomic position and grip strength at age 46 in the BCS70 excluding those participants unable to complete the grip strength assessments for health reasons (linear regression models in A) Females (n=3,872) and B) Males (n=3,675)) *[Sensitivity analysis]*

Table S9: Associations between indicators childhood and adulthood socioeconomic position and grip strength at age 46 in the BCS70 excluding those participants classified as severely hampered according to the European Statistics of Income and Living Conditions (EU-SILC) classification disability definition or with missing disability data (linear regression models in A) Females (n=3,638) and B) Males (n=3,394)) *[Sensitivity analysis]*

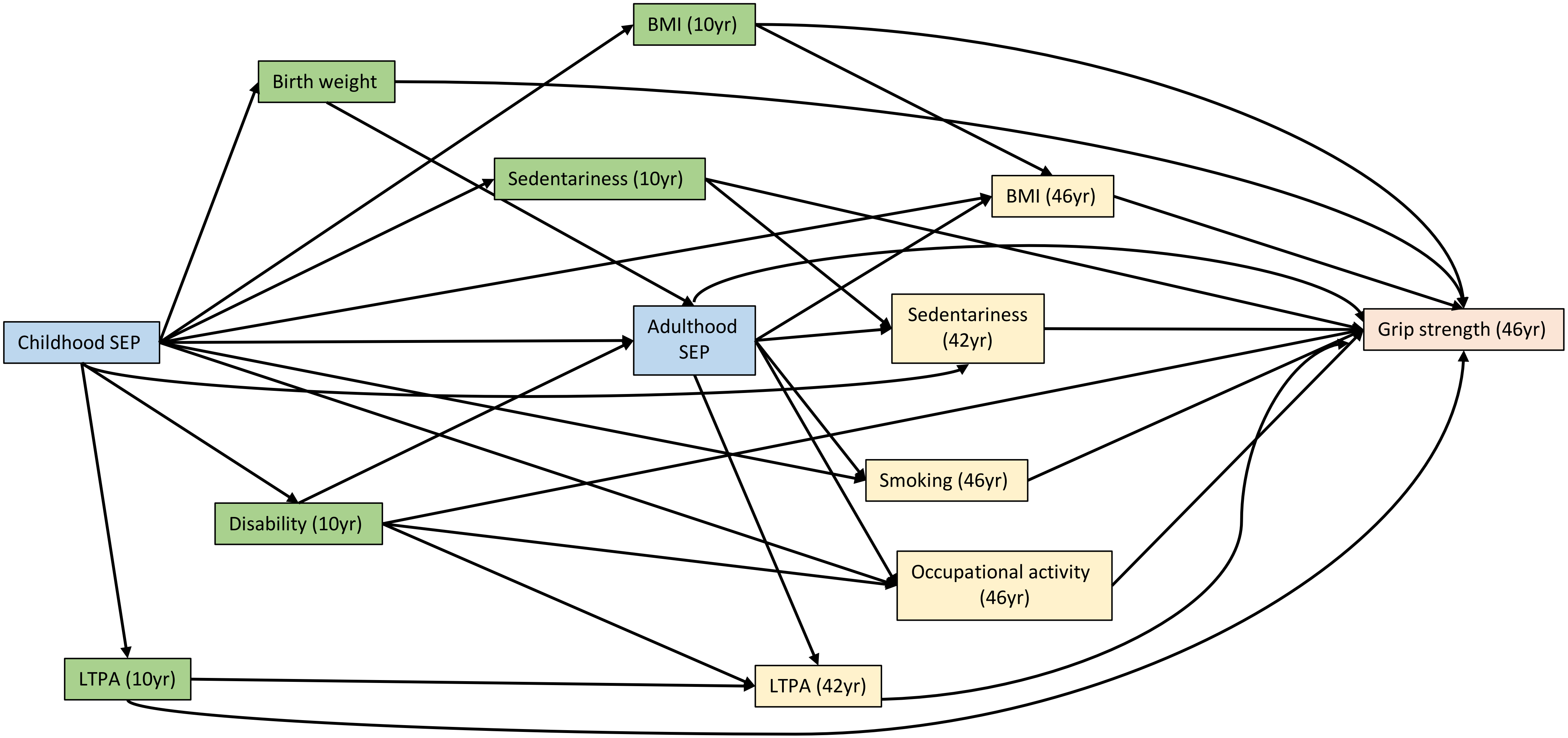

Figure S1: Pathway diagram detailing the proposed pathways of associations. For the relationships between indicators of childhood SEP and grip strength (age 46y), adulthood SEP and all the covariates in the green and yellow boxes are treated as potential mediators. For the relationships between indicators of adulthood SEP and grip strength, childhood SEP and the covariates in the green boxes are treated as potential confounders, while the covariates in the yellow boxes are treated as potential mediators.

Table S1: Variables used in multiple imputation

Variable	Type of variable	Method used to predict missing data for this variable	N (%)* with observed data on this variable
**Exposure variable**			
Father's occupational class at age 5y	Ordinal	Ordered logistic regression	7,198 (94.5%)
Mother's highest qualification at age 5y	Ordinal	Ordered logistic regression	5,931 (77.9%)
Father's highest qualification at age 5y	Ordinal	Ordered logistic regression	5,565 (73.1%)
Own occupational class at age 46y	Ordinal	Ordered logistic regression	6,404 (84.1%)
Own highest qualification at age 46y	Ordinal	Ordered logistic regression	7,512 (98.6%)
**Covariates**			
Birth weight	Continuous	Predictive mean matching	7,046 (92.5%)
Body Mass Index at age 10y	Continuous	Predictive mean matching	6,016 (79.0%)
Leisure-time physical activity at age 10y	Ordinal	Ordered logistic regression	6,607 (86.7%)
Sedentary behaviour at age 10y	Ordinal	Ordered logistic regression	6,626 (87.0%)
Disability at age 10y	Ordinal	Ordered logistic regression	6,606 (86.7%)
Height at age 46y	Continuous	Predictive mean matching	7,553 (99.2%)
Body Mass Index at age 46y	Continuous	Predictive mean matching	7,387 (97.0%)
Smoking at age 42y	Ordinal	Ordered logistic regression	7,111 (93.4%)
Sedentary behaviour at age 42y	Ordinal	Ordered logistic regression	6,368 (83.6%)
Leisure-time physical activity at age 42y	Ordinal	Ordered logistic regression	7,008 (92.0%)
Occupational activity at age 46y	Ordinal	Ordered logistic regression	6,291 (82.6%)
**Auxiliary variables**			
Father's age at completion of education	Continuous	Predictive mean matching	6,781 (89.0%)
Mother's age at completion of education	Continuous	Predictive mean matching	7,007 (92.0%)
Mother's age at birth	Continuous	Predictive mean matching	7,012 (92.1%)
Mother's marital status at birth	Ordinal	Ordered logistic regression	7,045 (92.5%)
Number of older siblings at birth	Continuous	Predictive mean matching	7,052 (92.6%)
Father's occupational class at age 16y	Ordinal	Ordered logistic regression	6,798 (89.2%)

* For the percentage reported, the numerator is the observed data divided by the denominator (total analytic sample size: n=7,617)

Table S2: A comparison of the distributions of variables included in the multiple imputation models by completeness of data

Characteristics	Total N [a]	Mean (SD) or N (%) [a] No missing data [b] (N=3,075)	Incomplete data [c] (N=4,542)	p-value [d]
**Exposure variables**				
Father's occupational class at age 5y	7,198			<0.001
I Professional/II Intermediate		1,040 (33.8)	1,058 (25.7)	
III Skilled non-manual		306 (10.0)	483 (11.7)	
III Skilled manual		1,301 (42.3)	1,762 (42.7)	
IV Partly skilled/V Unskilled		428 (13.9)	820 (19.9)	
Mother's highest qualification at age 5y	5,931			0.007
Higher vocational/degree and higher		322 (10.5)	243 (8.5)	
A-level/equivalent		146 (4.7)	126 (4.4)	
Vocational/O-level/equivalent		1,116 (36.3)	989 (34.6)	
No qualification		1,491 (48.5)	1,498 (52.5)	
Father's highest qualification at age 5y	5,565			0.009
Higher vocational/degree and higher		600 (19.5)	432 (17.3)	
A-level/equivalent		278 (9.0)	196 (7.9)	
Vocational/O-level/equivalent		915 (29.8)	719 (28.9)	
No qualification		1,282 (41.7)	1,143 (45.9)	
Own occupational class at age 46y	6,404			<0.001
I Professional/II Intermediate		1,510 (49.1)	1,514 (45.5)	
III Skilled non-manual		724 (23.5)	742 (22.3)	
III Skilled manual		572 (18.6)	713 (21.4)	
IV Partly skilled/V Unskilled		269 (8.7)	360 (10.8)	
Own highest qualification at age 46y	7,512			<0.001
Degree and higher		915 (29.8)	1,063 (24.0)	
A-level and vocational qualification		490 (15.9)	585 (13.2)	
GCSEs		976 (31.7)	1,386 (31.2)	
No qualification		694 (22.6)	1,403 (31.6)	
**Covariates**				
Birth weight (kg) (Mean (SD))	7,046	3.34 (0.5)	3.29 (0.5)	<0.001
BMI (kg/m^2) at age 10y (Mean (SD))	6,016	16.78 (2.0)	16.89 (2.1)	0.044
Leisure-time physical activity at age 10y	6,607			0.400
Never or hardly ever		228 (7.4)	295 (8.4)	
Sometimes		1,201 (39.1)	1,362 (38.6)	
Often		1,646 (53.5)	1,875 (53.1)	

Characteristics	Total N [a]	Mean (SD) or N (%) [a]<br>No missing data [b]<br>(N=3,075)	Incomplete data [c]<br>(N=4,542)	p-value [d]
Sedentary behaviour (TV watching) at age 10y	6,626			0.900
Never or hardly ever		33 (1.1)	42 (1.2)	
Sometimes		608 (19.8)	714 (20.1)	
Often		2,434 (79.2)	2,795 (78.7)	
Disability at age 10y	6,606			0.031
No		2,887 (93.9)	3,259 (92.3)	
Yes, slight		175 (5.7)	248 (7.0)	
Yes, severe		13 (0.4)	24 (0.7)	
Height (m) at age 46y (Mean (SD))	7,553	1.71 (0.09)	1.69 (0.09)	0.002
BMI (kg/m^2) at age 46y (Mean (SD))	7,387	28.24 (5.3)	28.56 (5.6)	0.013
Smoking status at age 42y	7,111			<0.001
Never smoker		1,568 (51.0)	1,798 (44.5)	
Ex-smoker		900 (29.3)	1,177 (29.2)	
Current smoker (less than daily)		175 (5.7)	218 (5.4)	
Current smoker (daily)		432 (14.0)	843 (20.9)	
Sedentary behaviour (TV watching) at age 42y	6,368			<0.001
0 to <1 hour		481 (17.2)	590 (16.5)	
1 to <3 hours		1,718 (61.5)	2,048 (57.3)	
3 to <5 hours		477 (17.1)	699 (19.6)	
5+ hours		117 (4.2)	238 (6.7)	
Leisure-time physical activity (days/week) at age 42y	7,008			<0.001
0 days		761 (24.7)	1,183 (30.1)	
1 day per a week		380 (12.4)	448 (11.4)	
2 days per a week		503 (16.4)	539 (13.7)	
3 days per a week		477 (15.5)	563 (14.3)	
4/5 days per a week		557 (18.1)	653 (16.6)	
6/7 days per a week		397 (12.9)	547 (13.9)	
Occupational activity at age 46y	6,291			0.050
Sitting occupation		1,735 (56.4)	1,703 (53.0)	
Standing occupation		460 (15.0)	512 (15.9)	
Physical work		743 (24.2)	840 (26.1)	
Heavy manual work		137 (4.5)	161 (5.0)	
**Auxiliary variables**				
Father's age (y) at completion of education	6,781 [a]	16.36 (4.2)	16.22 (4.8)	0.200
Mother's age (y) at completion of education	7,007	15.99 (3.2)	15.88 (4.4)	0.200
Mother's age at birth (y)	7,012	26.21 (5.3)	25.88 (5.4)	0.010

Characteristics	Total N [a]	Mean (SD) or N (%) [a] No missing data [b] (N=3,075)	Incomplete data [c] (N=4,542)	p-value [d]
Mother's marital status at birth	7,045			<0.001
Married		2,994 (97.4)	3,676 (92.5)	
Separated		15 (0.5)	64 (1.6)	
Divorced		4 (0.1)	18 (0.5)	
Widowed		0 (0.0)	5 (0.1)	
Single		60 (2.0)	209 (5.3)	
Number of older siblings at birth	7,052	0.94 (1.2)	1.05 (1.3)	<0.001
Father's occupational class at age 16y	6,798			0.005
I Professional/II Intermediate		1,112 (36.8)	1,244 (33.0)	
III Skilled non-manual		329 (10.9)	445 (11.8)	
III Skilled manual		1,172 (38.7)	1,499 (39.7)	
IV Partly skilled/V Unskilled		412 (13.6)	585 (15.5)	

[a] Ns presented vary due to missing data

[b] Those participants in the main analytic sample (N=7'617) who have complete data for all key variables (father's occupational class at age 5y, mother's highest qualification at age 5y, father's highest qualifications at age 5y, own occupational class at age 46y, own highest qualification at age 46y, birth weight, BMI at age 10y, leisure-time physical activity at age 10y, sedentary behaviour at age 10y, disability at age 10y, height at age 46y, BMI at age 46y, smoking status at age 42y, sedentary behaviour at age 42y, leisure-time physical activity at age 42y, occupational activity at age 46y and grip strength at age 46).

[c] Those participants in the main analytic sample who have missing data on at least one key variable (father's occupational class at age 5y, mother's highest qualification at age 5y, father's highest qualifications at age 5y, own occupational class at age 46y, own highest qualification at age 46y, birth weight, BMI at age 10y, leisure-time physical activity at age 10y, sedentary behaviour at age 10y, disability at age 10y, height at age 46y, BMI at age 46y, smoking status at age 42y, sedentary behaviour at age 42y, leisure-time physical activity at age 42y and/or occupational activity at age 46y).

[d] p-values from chi-squared or t-tests comparing the two groups, as appropriate

Table S3: Associations between indicators of childhood socioeconomic position and grip strength at age 46 years in the BCS70 (linear regression models in A) Females (n=3,922) and B) Males (n=3,695))

	Model 1		Model 2		Model 3		Model 4		Model 5	
Socioeconomic indicators	Coefficient [a]	95% CI [b]	Coefficient [a]	95% CI [b]	Coefficient [a]	95% CI [b]	Coefficient [a]	95% CI [b]	Coefficient [a]	95% CI [b]
**Father's occupational class at age 5**										
A) Female										
I Professional/II Intermediate	0.00 (ref)	—	0.00 (ref)	—	0.00 (ref)	—	0.00 (ref)	—	0.00 (ref)	—
III Skilled non-manual	-0.54	-1.21, 0.13	-0.39	-1.03, 0.26	-0.42	-1.06, 0.23	-0.37	-1.01, 0.27	-0.33	-0.97, 0.31
III Skilled manual	-1.30	-1.75, -0.85	-0.87	-1.31, -0.44	-0.88	-1.31, -0.44	-0.82	-1.26, -0.38	-0.74	-1.19, -0.29
IV Partly skilled/V Unskilled	-1.42	-1.97, -0.87	-0.84	-1.37, -0.30	-0.77	-1.31, -0.24	-0.74	-1.28, -0.20	-0.64	-1.19, -0.08
p-values (overall [c]; test for trend [d])		<0.001; <0.001		<0.001; <0.001		<0.001; <0.001		<0.01; <0.001		0.01; <0.01
B) Male										
I Professional/II Intermediate	0.00 (ref)	—	0.00 (ref)	—	0.00 (ref)	—	0.00 (ref)	—	0.00 (ref)	—
III Skilled non-manual	-0.72	-1.77, 0.33	-0.64	-1.65, 0.37	-0.60	-1.60, 0.40	-0.62	-1.59, 0.35	-0.73	-1.70, 0.25
III Skilled manual	-0.08	-0.78, 0.63	0.46	-0.23, 1.15	0.47	-0.22, 1.15	-0.29	-0.97, 0.40	-0.42	-1.12, 0.27
IV Partly skilled/V Unskilled	-0.68	-1.59, 0.23	0.06	-0.83, 0.95	0.05	-0.83, 0.94	-0.65	-1.53, 0.23	-0.77	-1.66, 0.12
p-values (overall [c]; test for trend [d])		0.29; 0.35		0.14; 0.33		0.15; 0.34		0.42; 0.20		0.28; 0.11
**Mother's highest qualification at age 5**										
A) Female										
Vocational/degree and higher	0.00 (ref)	—	0.00 (ref)	—	0.00 (ref)	—	0.00 (ref)	—	0.00 (ref)	—
A-level/equivalent	-0.48	-1.64, 0.69	-0.55	-1.67, 0.56	-0.50	-1.61, 0.61	-0.44	-1.55, 0.67	-0.39	-1.50, 0.73
Vocational/O-level/equivalent	-0.84	-1.56, -0.11	-0.68	-1.38, 0.03	-0.70	-1.40, 0.00	-0.73	-1.42, -0.04	-0.63	-1.33, 0.08
No qualification	-1.46	-2.14, -0.78	-1.01	-1.67, -0.35	-1.00	-1.66, -0.35	-0.99	-1.65, -0.33	-0.84	-1.53, -0.16
p-values (overall [c]; test for trend [d])		<0.001; <0.001		<0.01; 0.01		0.02; <0.01		0.02; <0.01		0.09; 0.01
B) Male										
Vocational/degree and higher	0.00 (ref)	—	0.00 (ref)	—	0.00 (ref)	—	0.00 (ref)	—	0.00 (ref)	—
A-level/equivalent	-0.65	-2.40, 1.10	-0.57	-2.27, 1.12	-0.46	-2.15, 1.23	-1.15	-2.81, 0.52	-1.28	-2.94, 0.38
Vocational/O-level/equivalent	0.50	-0.61, 1.62	0.88	-0.21, 1.97	0.94	-0.15, 2.03	0.36	-0.71, 1.43	0.14	-0.94, 1.23
No qualification	0.17	-0.89, 1.24	0.97	-0.07, 2.01	1.03	-0.01, 2.07	0.02	-1.02, 1.06	-0.27	-1.33, 0.78
p-values (overall [c]; test for trend [d])		0.45; 0.70		0.07; 0.03		0.06; 0.02		0.24; 0.75		0.24; 0.80
**Father's highest qualification at age 5**										
A) Female										
Vocational/degree and higher	0.00 (ref)	—	0.00 (ref)	—	0.00 (ref)	—	0.00 (ref)	—	0.00 (ref)	—
A-level/equivalent	-0.22	-1.03, 0.60	0.08	-0.72, 0.87	0.05	-0.74, 0.83	0.07	-0.70, 0.85	0.15	-0.63, 0.93
Vocational/O-level/equivalent	-0.58	-1.18, 0.02	-0.34	-0.92, 0.24	-0.35	-0.93, 0.22	-0.32	-0.90, 0.25	-0.23	-0.81, 0.35
No qualification	-1.00	-1.55, -0.45	-0.50	-1.03, 0.04	-0.54	-1.07, 0.00	-0.45	-1.00, 0.10	-0.31	-0.87, 0.26
p-values (overall [c]; test for trend [d])		<0.01; <0.001		0.18; 0.04		0.15; 0.03		0.28; 0.06		0.54; 0.19
B) Male										
Vocational/degree and higher	0.00 (ref)	—	0.00 (ref)	—	0.00 (ref)	—	0.00 (ref)	—	0.00 (ref)	—
A-level/equivalent	0.13	-1.20, 1.45	0.40	-0.88, 1.67	0.26	-1.01, 1.53	-0.22	-1.47, 1.03	-0.36	-1.62, 0.89
Vocational/O-level/equivalent	0.44	-0.51, 1.38	0.70	-0.22, 1.61	0.62	-0.30, 1.53	-0.10	-1.00, 0.79	-0.27	-1.18, 0.64

No qualification	0.63	-0.23, 1.49	1.22	0.37, 2.07	1.19	0.35, 2.04	-0.12	-0.97, 0.74	-0.31	-1.19, 0.57
p-values (overall [c]; test for trend [d])		0.45; 0.12		0.03; <0.01		0.03; <0.01		0.93; 0.74		0.85; 0.55

Note 1: results are combined from analyses run across 50 imputed datasets.

Note 2: sample was restricted to those with valid measures of grip strength at age 46 years (including those 70 individuals unable for health reasons with imputed values).

Model 1: unadjusted (p-values from formal tests of sex interaction, *p*=0.01 for Fathers occupational class at age 5y, p=0.03 for Mother's highest qualification at age 5y and p=0.01 for Father's highest qualification at age 5y).

Model 2: adjusted for height at age 46y.

Model 3: adjusted for Model 2 + birth weight (kg), BMI at age 10y (kg/m^2), leisure-time physical activity at age 10y, sedentary behaviour (TV watching) at age 10y and disability at age 10y,

Model 4: adjusted for Model 3 + BMI at age 46y (kg/m^2) + smoking status at age 42y, sedentary behaviour (TV watching) at age 42y, leisure-time physical activity (days/week) at age 42y and occupational activity at age 46y.

Model 5: adjusted for Model 4 + own occupation at age 46y + own highest qualification at age 46y.

[a] Coefficient: Difference in mean grip strength (kg).

[b] CI: Confidence Interval.

[c] P-value: p-value from a likelihood ratio test of the overall association.

[d] P-trend: p-value not presented for relationship where there was significant deviation from linearity.

Table S4: Associations between indicators of adulthood socioeconomic position and grip strength at age 46 years in the BCS70 (linear regression models in A) Females (n=3,922) and B) Males (n=3,695))

	Model 1		Model 2		Model 3		Model 4	
Socioeconomic indicators	Coefficient [a]	95% CI [b]	Coefficient [a]	95% CI [b]	Coefficient [a]	95% CI [b]	Coefficient [a]	95% CI [b]
**Own occupational class at age 46y**								
A) Female								
I Professional/II Intermediate	0.00 (ref)	—	0.00 (ref)	—	0.00 (ref)	—	0.00 (ref)	—
III Skilled non-manual	-0.78	-1.23, -0.32	-0.57	-1.01, -0.13	-0.44	-0.88, 0.00	-0.33	-0.77, 0.11
III Skilled manual	-0.50	-1.20, 0.20	-0.20	-0.88, 0.47	-0.17	-0.84, 0.51	-0.31	-1.01, 0.39
IV Partly skilled/V Unskilled	-0.92	-1.56, -0.27	-0.53	-1.16, 0.09	-0.49	-1.10, 0.12	-0.64	-1.33, 0.05
p-values (overall [c]; test for trend [d])		<0.01; <0.01		0.05; 0.08		0.14; 0.12		0.18; 0.06
B) Male								
I Professional/II Intermediate	0.00 (ref)	—	0.00 (ref)	—	0.00 (ref)	—	0.00 (ref)	—
III Skilled non-manual	-0.76	-1.70, 0.17	-0.66	-1.57, 0.25	-0.57	-1.48, 0.33	-0.84	-1.73, 0.05
III Skilled manual	1.33	0.60, 2.06	1.88	1.17, 2.59	1.92	1.21, 2.62	0.01	-0.85, 0.88
IV Partly skilled/V Unskilled	-0.64	-1.81, 0.54	-0.02	-1.16, 1.12	0.23	-0.91, 1.37	-1.25	-2.50, 0.00
p-values (overall [c]; test for trend [d])		<0.001; -		<0.001; -		<0.001; -		0.06; 0.17
**Own highest qualification at age 46y**								
A) Female								
Degree and higher	0.00 (ref)	—	0.00 (ref)	—	0.00 (ref)	—	0.00 (ref)	—
Advance and vocational qualification	-0.63	-1.21, -0.05	-0.43	-0.99, 0.13	-0.50	-1.05, 0.06	-0.62	-1.18, -0.05
GCSEs	-0.65	-1.13, -0.17	-0.29	-0.75, 0.18	-0.25	-0.71, 0.21	-0.29	-0.77, 0.18
No qualification	-1.25	-1.76, -0.75	-0.80	-1.29, -0.31	-0.74	-1.23, -0.25	-0.76	-1.28, -0.24
p-values (overall [c]; test for trend [d])		<0.001; <0.001		0.01; <0.01		<0.02; 0.01		0.02; 0.2
Male								
Degree and higher	0.00 (ref)	—	0.00 (ref)	—	0.00 (ref)	—	0.00 (ref)	—
A-level and vocational qualification	1.46	0.46, 2.46	1.76	0.79, 2.73	1.67	0.71, 2.64	1.12	0.17, 2.08
GCSEs	1.68	0.89, 2.46	2.16	1.40, 2.92	2.18	1.43, 2.94	0.97	0.16, 1.77
No qualification	1.59	0.81, 2.36	2.26	1.50, 3.02	2.34	1.58, 3.10	1.12	0.29, 1.95
p-values (overall [c]; test for trend [d])		<0.001; -		<0.001; -		<0.001; -		0.03; 0.02

Note 1: results are combined from analyses run across 50 imputed datasets.

Note 2: sample was restricted to those with valid measures of grip strength at age 46 years (including those 70 individuals unable for health reasons with imputed values).

Model 1: unadjusted (p-values from formal tests of sex interaction, p<0.01 for own occupation at age 46y, and p<0.001 for own highest qualification at age 46y)

Model 2: adjusted for height at age 46y.

Model 3: adjusted for Model 2 + birth weight (kg), BMI at age 10y (kg/m^2), leisure-time physical activity at age 10y, sedentary behaviour (TV watching) at age 10y and disability at age 10y,

Model 4: adjusted for Model 3 + BMI at age 46y (kg/m^2) + smoking status at age 42y, sedentary behaviour (TV watching) at age 42y, leisure-time physical activity (days/week) at age 42y and occupational activity at age 46y.

Model 5: adjusted for Model 4 + own occupation at age 46y + own highest qualification at age 46y.

[a] Coefficient: Difference in mean grip strength (kg).

[b] CI: Confidence Interval.

[c] P-value: p-value from a likelihood ratio test of the overall association.

[d] P-trend: p-value not presented for relationship where there was significant deviation from linearity.

Table S5: Associations between adulthood socioeconomic position and grip strength at age 46 years in the BCS70 with individual adjustments for each adult covariate (linear regression models in Males (n=3,695))

	Model 4a	Model 4b	Model 4c	Model 4d	Model 4e
Occupational class[1]	Coefficient [a] (95% CI [b])	Coefficient [a] (95% CI [b])	Coefficient [a] (95% CI [b])	Coefficient [a] (95% CI [b])	Coefficient [a] (95% CI [b])
**Own occupational class at age 46y**					
I Professional/II Intermediate	0.00 (ref)	0.00 (ref)	0.00 (ref)	0.00 (ref)	0.00 (ref)
III Skilled non-manual	-0.71 (-1.61, 0.19)	-0.56 (-1.46, 0.35)	-0.43 (-1.33, 0.48)	-0.62 (-1.52, 0.28)	-0.83 (-1.74, 0.07)
III Skilled manual	1.83 (1.13, 2.53)	2.00 (1.28, 2.72)	2.17 (1.45, 2.89)	1.86 (1.15, 2.57)	-0.26 (-1.13, 0.62)
IV Partly skilled/V Unskilled	0.27 (-0.86, 1.39)	0.33 (-0.83, 1.48)	0.78 (-0.39, 1.96)	0.01 (-1.13, 1.15)	-1.84 (-3.08, -0.60)
p-values (overall [c]; test for trend [d])	<0.001; -	<0.001; -	<0.001; -	<0.001; -	0.02; 0.02
**Own highest qualification at age 46y**					
Degree and higher	0.00 (ref)	0.00 (ref)	0.00 (ref)	0.00 (ref)	0.00 (ref)
A-level and vocational qualification	1.39 (0.43, 2.35)	1.71 (0.74, 2.67)	1.82 (0.86, 2.79)	1.66 (0.70, 2.62)	1.28 (0.32, 2.24)
GCSEs	1.81 (1.05, 2.57)	2.25 (1.49, 3.02)	2.44 (1.67, 3.21)	2.16 (1.40, 2.92)	1.11 (0.31, 1.90)
No qualification	2.04 (1.29, 2.80)	2.45 (1.68, 3.23)	2.71 (1.93, 3.49)	2.34 (1.58, 3.11)	1.01 (0.19, 1.82)
p-values (overall [c]; test for trend [d])	<0.001; <0.001	<0.001; -	<0.001; -	<0.001; -	0.02; 0.03

Note 1: results are combined from analyses run across 50 imputed datasets.
Note 2: sample was restricted to those with valid measures of grip strength at age 46 years (including those 70 individuals unable for health reasons with imputed values).
Model 3: adjusted for height at age 46y + birth weight (kg), BMI at age 10y (kg/m^2), leisure-time physical activity at age 10y, sedentary behaviour (TV watching) at age 10y and disability at age 10y.

Model 4a: adjusted for Model 3 + BMI at age 46y (kg/m^2).

Model 4b: adjusted for Model 3 + smoking status at age 42y.

Model 4c: adjusted for Model 3 + sedentary behaviour (TV watching) at age 42y.

Model 4d: adjusted for Model 3 + leisure-time physical activity at age 42y.

Model 4e: adjusted for Model 3 + occupational activity at age 46y.

[a] Coefficient: Difference in mean grip strength (kg).

[b] CI: Confidence Interval.

[c] P-value: p-value from a likelihood ratio test of the overall association.

[d] P-trend: p-value not presented for relationship where there was significant deviation from linearity.

Table S6: Unadjusted associations between indicators of childhood and adulthood socioeconomic position and grip strength at age 46 years in the BCS70 on observed data (linear regression models in A) Females (n=3,922) and B) Males (n=3,695))

	A) Female			B) Male		
Socioeconomic indicators	N	Coefficient [a]	95% CI [b]	N	Coefficient [a]	95% CI [b]
**Father's occupational class at age 5y**	3,728			3,470		
I Professional/II Intermediate		0.00 (ref)	—		0.00 (ref)	—
III Skilled non-manual		-0.57	-1.23, 0.10		-0.66	-1.71, 0.39
III Skilled manual		-1.32	-1.78, -0.87		-0.06	-0.77, 0.66
IV Partly skilled/V Unskilled		-1.42	-1.98, -0.87		-0.64	-1.56, 0.29
p-values (overall [c]; test for trend [d])		3,728	<0.001; <0.001			0.36; 0.40
**Mother's highest qualification at age 5y**	3,079			2,852		
Vocational/degree and higher		0.00 (ref)	—		0.00 (ref)	—
A-level/equivalent		-0.31	-1.47, 0.86		-0.93	-2.81, 0.94
Vocational/O-level/equivalent		-0.82	-1.56, -0.07		0.33	-0.88, 1.54
No qualification		-1.46	-2.18, -0.74		-0.06	-1.24, 1.11
p-values (overall[3]; test for trend[4])			<0.001; <0.001			0.43; 0.99
**Father's highest qualification at age 5y**	2,870			2,695		
Vocational/degree and higher		0.00 (ref)	—		0.00 (ref)	—
A-level/equivalent		-0.17	-1.05, 0.71		0.00	-1.43, 1.43
Vocational/O-level/equivalent		-0.51	-1.14, 0.13		0.37	-0.64, 1.38
No qualification		-0.98	-1.57, -0.38		0.68	-0.26, 1.62
p-values (overall [c]; test for trend [d])			<0.01; <0.001			0.47; 0.12
**Own occupational class at age 46y**	2,870			2,695		
I Professional/II Intermediate		0.00 (ref)	—		0.00 (ref)	—
III Skilled non-manual		-0.64	-1.10, -0.19		-0.56	-1.46, 0.33
III Skilled manual		-0.06	-0.74, 0.61		1.90	1.21, 2.60
IV Partly skilled/V Unskilled		-0.49	-1.13, 0.15		0.35	-0.79, 1.49
p-values (overall [c]; test for trend [d])			0.03; -			<0.001; -
**Own highest qualification at age 46y**	2,870			2,695		
Degree and higher		0.00 (ref)	—		0.00 (ref)	—
A-level and vocational qualification		-0.64	-1.22, -0.06		1.48	0.48, 2.48
GCSEs		-0.66	-1.13, -0.18		1.71	0.93, 2.49
No qualification		-1.26	-1.77, -0.76		1.63	0.85, 2.41
p-values (overall [c]; test for trend [d])			<0.01; <0.001			<0.001; -

Note 1: results are combined from analyses run across 50 imputed datasets.

Note 2: sample was restricted to those with valid measures of grip strength at age 46 years (including those 70 individuals unable for health reasons with imputed values)

Note 3: results displayed for unadjusted model (p-values from formal tests of sex interaction, p=0.01 for fathers occupational class at age 5y, p=0.05 for mother's highest qualification at age 5y, p=0.01 for father's highest qualification at age 5y; p<0.01 for own occupational class at age 46y and p<0.001 for own highest qualification at age 46y). [*]70 participants unable to complete the grip strength tests for health reasons were included by allocating them grip strength values equivalent to the mean of the bottom sex-specific fifth.

[a] Coefficient: Difference in mean grip strength (kg); [b] CI: Confidence Interval; [c] P-value: p-value from a likelihood ratio test of the overall association; [d] P-trend: p-value not presented for relationship where there was significant deviation from linearity.

Table S7: Associations between indicators of childhood and adulthood socioeconomic position and grip strength at age 46 years in the BCS70 on the sample who completed the grip strength assessment standing unsupported (linear regression models in A) Females (n=3,498) and B) Males (n=3,392))

	A) Female				B) Male			
	Model 1		Model 4		Model 1		Model 4	
Socioeconomic indicators	Coefficient [a]	95% CI [b]	Coefficient [a]	95% CI [b]	Coefficient [a]	95% CI [b]	Coefficient [a]	95% CI [b]
**Father's occupational class at age 5**								
I Professional/II Intermediate	0.00 (ref)	—	0.00 (ref)	—	0.00 (ref)	—	0.00 (ref)	—
III Skilled non-manual	-0.37	-1.05, 0.31	-0.24	-0.89, 0.41	-0.74	-1.82, 0.33	-0.70	-1.70, 0.29
III Skilled manual	-1.06	-1.52, -0.59	-0.69	-1.15, -0.24	-0.12	-1.30, 0.60	-0.37	-1.07, 0.33
IV Partly skilled/V Unskilled	-1.22	-1.79, -0.65	-0.59	-1.15, -0.03	-0.65	-1.58, 0.28	-0.66	-1.55, 0.24
p-values (overall [c]; test for trend [d])		<0.001; <0.001		0.02; <0.01		0.36; 0.35		0.39; 0.18
**Mother's highest qualification at age 5**								
Vocational/degree and higher	0.00 (ref)	—	0.00 (ref)	—	0.00 (ref)	—	0.00 (ref)	—
A-level/equivalent	-0.55	-1.75, 0.65	-0.54	-1.68, 0.59	-0.43	-2.23, 1.36	-1.00	-2.70, 0.71
Vocational/O-level/equivalent	-0.83	-1.57, -0.09	-0.76	-1.47, -0.06	0.72	-0.41, 1.85	0.46	-0.62, 1.53
No qualification	-1.35	-2.04, -0.65	-0.98	-1.66, -0.31	0.24	-0.84, 1.31	-0.05	-1.10, 1.00
p-values (overall [c]; test for trend [d])		<0.001; <0.001		0.03; <0.01		0.31; 0.74		0.21; 0.98
**Father's highest qualification at age 5**								
Vocational/degree and higher	0.00 (ref)	—	0.00 (ref)	—	0.00 (ref)	—	0.00 (ref)	—
A-level/equivalent	0.05	-0.81, 0.91	0.30	-0.51, 1.12	0.28	-1.08, 1.63	-0.05	-1.33, 1.23
Vocational/O-level/equivalent	-0.45	-1.09, 0.19	-0.23	-0.84, 0.38	0.46	-0.50, 1.42	-0.11	-1.03, 0.80
No qualification	-0.72	-1.29, -0.14	-0.30	-0.87, 0.28	0.67	-0.22, 1.55	-0.11	-1.00, 0.78
p-values (overall [c]; test for trend [d])		0.04; <0.01		0.37; 0.17		0.49; 0.13		0.96; 0.81
**Own occupational class at age 46y**								
I Professional/II Intermediate	0.00 (ref)	—	0.00 (ref)	—	0.00 (ref)	—	0.00 (ref)	—
III Skilled non-manual	-0.78	-1.25, -0.30	-0.43	-0.89, 0.03	-0.66	-1.61, 0.29	-0.82	-1.72, 0.09
III Skilled manual	-0.36	-1.08, 0.37	-0.26	-0.99, 0.47	1.47	0.74, 2.20	0.13	-0.73, 0.99
IV Partly skilled/V Unskilled	-0.62	-1.28, 0.04	-0.45	-1.16, 0.26	-0.71	-1.91, 0.49	-1.36	-2.64, -0.07
p-values (overall [c]; test for trend [d])		<0.01; -		0.21; 0.15		<0.01; -		0.03; -
**Own highest qualification at age 46y**								
Degree and higher	0.00 (ref)	—	0.00 (ref)	—	0.00 (ref)	—	0.00 (ref)	—
A-level and vocational qualification	-0.67	-1.27, -0.07	-0.67	-1.24, -0.09	1.61	0.59, 2.64	1.25	0.28, 2.22
GCSEs	-0.60	-1.10, -0.11	-0.31	-0.80, 0.18	1.65	0.85, 2.45	0.86	0.03, 1.68
No qualification	-0.81	-1.34, -0.29	-0.42	-0.96, 0.12	1.79	1.00, 2.58	1.25	0.41, 2.09
p-values (overall [c]; test for trend [d])		<0.01; <0.01		0.14; 0.23		<0.001; <0.001		0.02; <0.01

Note 1: results are combined from analyses run across 50 imputed datasets.

Note 2: sample was restricted to those with valid measures of grip strength at age 46 years (including those 70 individuals unable for health reasons with imputed values).

Model 1: unadjusted (p-values from formal tests of sex interaction, $p<0.07$ for fathers occupational class at age 5y, $p<0.05$ for mother's highest qualification at age 5y, $p=0.06$ for father's highest qualification at age 5y, $p<0.01$ for own occupational class at age 46y and $p<0.001$ for own highest qualification at age 46y).

Model 4: adjusted for Model 3 + BMI at age 46y (kg/m^2) + smoking status at age 42y, sedentary behaviour (TV watching) at age 42y, leisure-time physical activity (days/week) at age 42y and occupational activity at age 46y.

[a] Coefficient: Difference in mean grip strength (kg).

[b] CI: Confidence Interval.

[c] P-value: p-value from a likelihood ratio test of the overall association.

[d] P-trend: p-value not presented for relationship where there was significant deviation from linearity.

Table S8: Associations between indicators of childhood and adulthood socioeconomic position and grip strength at age 46 in the BCS70 excluding those participants unable to complete the grip strength assessments for health reasons (linear regression models in A) Females (n=3,872) and B) Males (n=3,675))

	A) Female				B) Male			
	Model 1		Model 4		Model 1		Model 4	
Socioeconomic indicators	Coefficient [a]	95% CI [b]	Coefficient [a]	95% CI [b]	Coefficient [a]	95% CI [b]	Coefficient [a]	95% CI [b]
**Father's occupational class at age 5**								
I Professional/II Intermediate	0.00 (ref)	—	0.00 (ref)	—	0.00 (ref)	—	0.00 (ref)	—
III Skilled non-manual	-0.56	-1.23, 0.11	-0.40	-1.04, 0.24	-0.75	-1.80, 0.29	-0.65	-1.62, 0.32
III Skilled manual	-1.25	-1.70, -0.80	-0.81	-1.25, -0.37	-0.06	-0.77, 0.65	-0.29	-0.98, 0.39
IV Partly skilled/V Unskilled	-1.39	-1.94, -0.84	-0.74	-1.28, -0.19	-0.68	-1.59, 0.23	-0.66	-1.54, 0.21
p-values (overall [c]; test for trend [d])		<0.001; <0.001		<0.01; 0.001		0.26; 0.37		0.39; 0.19
**Mother's highest qualification at age 5**								
Vocational/degree and higher	0.00 (ref)	—	0.00 (ref)	—	0.00 (ref)	—	0.00 (ref)	—
A-level/equivalent	-0.52	-1.69, 0.64	-0.50	-1.60, 0.60	-0.67	-2.42, 1.07	-1.19	-2.84, 0.47
Vocational/O-level/equivalent	-0.79	-1.52, -0.06	-0.70	-1.40, -0.01	0.55	-0.57, 1.67	0.39	-0.67, 1.45
No qualification	-1.42	-2.10, -0.74	-1.00	-1.66, -0.34	0.21	-0.85, 1.27	0.04	-1.00, 1.07
p-values (overall [c]; test for trend [d])		<0.001; <0.001		0.02; <0.01		0.39; 0.64		0.19; 0.72
**Father's highest qualification at age 5**								
Vocational/degree and higher	0.00 (ref)	—	0.00 (ref)	—	0.00 (ref)	—	0.00 (ref)	—
A-level/equivalent	-0.20	-1.01, 0.62	0.10	-0.68, 0.88	0.16	-1.17, 1.49	-0.21	-1.46, 1.04
Vocational/O-level/equivalent	-0.52	-1.12, 0.08	-0.28	-0.86, 0.30	0.42	-0.53, 1.37	-0.13	-1.03, 0.76
No qualification	-0.94	-1.50, -0.39	-0.44	-0.99, 0.11	0.63	-0.23, 1.50	-0.14	-1.00, 0.72
p-values (overall [c]; test for trend [d])		<0.01; <0.001		0.30; 0.07		0.47; 0.13		0.93; 0.80
**Own occupational class at age 46y**								
I Professional/II Intermediate	0.00 (ref)	—	0.00 (ref)	—	0.00 (ref)	—	0.00 (ref)	—
III Skilled non-manual	-0.72	-1.18, -0.27	-0.30	-0.74, 0.15	-0.77	-1.70, 0.16	-0.86	-1.74, 0.02
III Skilled manual	-0.44	-1.15, 0.26	-0.28	-0.98, 0.42	1.37	0.64, 2.10	0.01	-0.85, 0.87
IV Partly skilled/V Unskilled	-0.82	-1.47, -0.17	-0.61	-1.30, 0.09	-0.59	-1.77, 0.58	-1.26	-2.51, -0.01
p-values (overall [c]; test for trend [d])		<0.01; <0.01		0.23; 0.08		<0.001; -		0.05; -
**Own highest qualification at age 46y**								
Degree and higher	0.00 (ref)	—	0.00 (ref)	—	0.00 (ref)	—	0.00 (ref)	—
A-level and vocational qualification	-0.58	-1.16, 0.00	-0.59	-1.15, -0.03	1.48	0.48, 2.48	1.12	0.17, 2.07
GCSEs	-0.57	-1.05, -0.10	-0.24	-0.71, 0.24	1.69	0.90, 2.47	0.96	0.15, 1.76
No qualification	-1.19	-1.69, -0.68	-0.74	-1.26, -0.21	1.67	0.89, 2.45	1.18	0.35, 2.01
p-values (overall [c]; test for trend [d])		<0.001; <0.001		0.02; 0.02		<0.001; <0.001		0.02; 0.01

Note 1: results are combined from analyses run across 50 imputed datasets.

Model 1: unadjusted (p-values from formal tests of sex interaction, $p<0.01$ for fathers occupation at age 5y, $p<0.03$ for mother's highest qualification at age 5y, $p=0.02$ for father's highest qualification at age 5y, $p<0.01$ for own occupational class at age 46y and $p<0.001$ for own highest qualification at age 46y).

Model 4: adjusted for Model 3 + BMI at age 46y (kg/m^2) + smoking status at age 42y, sedentary behaviour (TV watching) at age 42y, leisure-time physical activity (days/week) at age 42y and occupational activity at age 46y.

[a] Coefficient: Difference in mean grip strength (kg).

[b] CI: Confidence Interval.

[c] P-value: p-value from a likelihood ratio test of the overall association.

[d] P-trend: p-value not presented for relationship where there was significant deviation from linearity.

Table S9: Associations between indicators childhood and adulthood socioeconomic position and grip strength at age 46 in the BCS70 excluding those participants classified as severely hampered according to the European Statistics of Income and Living Conditions (EU-SILC) classification disability definition or with missing disability data (linear regression models in A) Females (n=3,638) and B) Males (n=3,394))

	A) Female				B) Male			
	Model 1		Model 4		Model 1		Model 4	
Socioeconomic indicators	Coefficient [a]	95% CI [b]	Coefficient [a]	95% CI [b]	Coefficient [a]	95% CI [b]	Coefficient [a]	95% CI [b]
**Father's occupational class at age 5**								
I Professional/II Intermediate	0.00 (ref)	—	0.00 (ref)	—	0.00 (ref)	—	0.00 (ref)	—
III Skilled non-manual	-0.38	-1.04, 0.29	-0.23	-0.87, 0.41	-0.72	-1.77, 0.32	-0.63	-1.59, 0.34
III Skilled manual	-1.00	-1.45, -0.56	-0.65	-1.08, -0.22	0.03	-0.67, 0.74	-0.30	-0.98, 0.38
IV Partly skilled/V Unskilled	-1.15	-1.70, -0.60	-0.59	-1.12, -0.05	-0.31	-1.23, 0.60	-0.52	-1.39, 0.36
p-values (overall [c]; test for trend [d])		<0.001; <0.001		0.02; <0.01		0.46; <0.83		0.52; 0.28
**Mother's highest qualification at age 5**								
Vocational/degree and higher	0.00 (ref)	—	0.00 (ref)	—	0.00 (ref)	—	0.00 (ref)	—
A-level/equivalent	-0.38	-1.55, 0.80	-0.38	-1.48, 0.73	-0.55	-2.27, 1.18	-1.15	-2.79, 0.48
Vocational/O-level/equivalent	-0.71	-1.43, 0.00	-0.66	-1.34, 0.02	0.61	-0.49, 1.71	0.40	-0.65, 1.44
No qualification	-1.43	-2.10, -0.75	-1.12	-1.76, -0.47	0.41	-0.64, 1.46	0.11	-0.91, 1.13
p-values (overall [c]; test for trend [d])		<0.001; <0.001		<0.01; <0.001		0.40; 0.37		0.20; 0.59
**Father's highest qualification at age 5**								
Vocational/degree and higher	0.00 (ref)	—	0.00 (ref)	—	0.00 (ref)	—	0.00 (ref)	—
A-level/equivalent	0.05	-0.74, 0.84	0.27	-0.48, 1.02	0.31	-1.03, 1.64	-0.09	-1.34, 1.17
Vocational/O-level/equivalent	-0.47	-1.05, 0.12	-0.29	-0.85, 0.27	0.50	-0.44, 1.43	-0.06	-0.96, 0.83
No qualification	-0.72	-1.26, -0.18	-0.36	-0.90, 0.18	0.90	0.04, 1.75	0.09	-0.76, 0.93
p-values (overall [c]; test for trend [d])		0.02; <0.01		0.24; 0.09		0.18; 0.03		0.94; 0.80
**Own occupational class at age 46y**								
I Professional/II Intermediate	0.00 (ref)	—	0.00 (ref)	—	0.00 (ref)	—	0.00 (ref)	—
III Skilled non-manual	-0.71	-1.15, -0.27	-0.36	-0.79, 0.07	-0.80	-1.70, 0.11	-1.01	-1.87, -0.15
III Skilled manual	-0.19	-0.87, 0.50	-0.12	-0.80, 0.56	1.53	0.83, 2.23	-0.07	-0.90, 0.76
IV Partly skilled/V Unskilled	-0.55	-1.18, 0.07	-0.45	-1.13, 0.22	-0.11	-1.25, 1.04	-1.23	-2.46, -0.01
p-values (overall [c]; test for trend [d])		0.01; -		0.27; 0.18		<0.01; -		0.03; -
**Own highest qualification at age 46y**								
Degree and higher	0.00 (ref)	—	0.00 (ref)	—	0.00 (ref)	—	0.00 (ref)	—
A-level and vocational qualification	-0.73	-1.30, -0.17	-0.79	-1.34, -0.25	1.58	0.59, 2.56	1.14	0.20, 2.08
GCSEs	-0.49	-0.96, -0.02	-0.29	-0.75, 0.18	1.75	0.97, 2.52	0.85	0.06, 1.65
No qualification	-0.82	-1.33, -0.32	-0.56	-1.07, -0.04	1.97	1.19, 2.75	1.18	0.36, 2.00
p-values (overall [c]; test for trend [d])		<0.01; <0.01		0.02; -		<0.001; <0.001		0.02; <0.01

Note 1: results are combined from analyses run across 50 imputed datasets.
Note 2: sample was restricted to those with valid measures of grip strength at age 46 years (including those 70 individuals unable for health reasons with imputed values).

Model 1: unadjusted (p-values from formal tests of sex interaction, p=0.03 for father's occupational class at age 5y, p<0.01 for mother's highest qualification at age 5y, p=0.01 for father's highest qualification at age 5y, p<0.01 for own occupational class at age 46y and p<0.001 for own highest qualification at age 46y).

Model 4: adjusted for Model 3 + BMI at age 46y (kg/m^2) + smoking status at age 42y, sedentary behaviour (TV watching) at age 42y, leisure-time physical activity (days/week) at age 42y and occupational activity at age 46y.

[a] Coefficient: Difference in mean grip strength (kg).

[b] CI: Confidence Interval.

[c] P-value: p-value from a likelihood ratio test of the overall association.

[d] P-trend: p-value not presented for relationship where there was significant deviation from linearity.
